# Metals on the Menu—Analyzing the Presence, Importance, and Consequences

**DOI:** 10.3390/foods13121890

**Published:** 2024-06-16

**Authors:** Vedran Milanković, Tamara Tasić, Andreja Leskovac, Sandra Petrović, Miloš Mitić, Tamara Lazarević-Pašti, Mirjana Novković, Nebojša Potkonjak

**Affiliations:** 1 VINČA Institute of Nuclear Sciences—National Institute of the Republic of Serbia, University of Belgrade, Mike Petrovica Alasa 12-14, 11000 Belgrade, Serbia; vedran.milankovic@vin.bg.ac.rs (V.M.); tamara.tasic@vin.bg.ac.rs (T.T.); andreja@vin.bg.ac.rs (A.L.); sandra@vin.bg.ac.rs (S.P.); milos.mitic@vin.bg.ac.rs (M.M.); tamara@vin.bg.ac.rs (T.L.-P.); 2 Group for Muscle Cellular and Molecular Biology, Institute of Molecular Genetics and Genetic Engineering, University of Belgrade, Vojvode Stepe 444a, 11000 Belgrade, Serbia; mirjananovkovic@imgge.bg.ac.rs

**Keywords:** alkali metals, alkaline earth metals, heavy metals, fruit, vegetables, meat, seafood, benefits, health issues

## Abstract

Metals are integral components of the natural environment, and their presence in the food supply is inevitable and complex. While essential metals such as sodium, potassium, magnesium, calcium, iron, zinc, and copper are crucial for various physiological functions and must be consumed through the diet, others, like lead, mercury, and cadmium, are toxic even at low concentrations and pose serious health risks. This study comprehensively analyzes the presence, importance, and consequences of metals in the food chain. We explore the pathways through which metals enter the food supply, their distribution across different food types, and the associated health implications. By examining current regulatory standards for maximum allowable levels of various metals, we highlight the importance of ensuring food safety and protecting public health. Furthermore, this research underscores the need for continuous monitoring and management of metal content in food, especially as global agricultural and food production practices evolve. Our findings aim to inform dietary recommendations, food fortification strategies, and regulatory policies, ultimately contributing to safer and more nutritionally balanced diets.

## 1. Introduction

Metals are omnipresent in our environment, finding their way into the food we consume through various natural and anthropogenic pathways [1,2]. Food is the major source of exposure to both essential and nonessential metals [3,4]. With their diverse properties and wide-ranging applications, they constitute a fundamental component of our natural environment and industrial processes. Metals can be classified based on different criteria, such as their density, abundance, toxicity, or roles in biological systems. Common categories are heavy metals, trace metals, alkali metals, alkaline earth metals, transition metals, noble metals, and radioactive metals [5].

**Figure 1:**
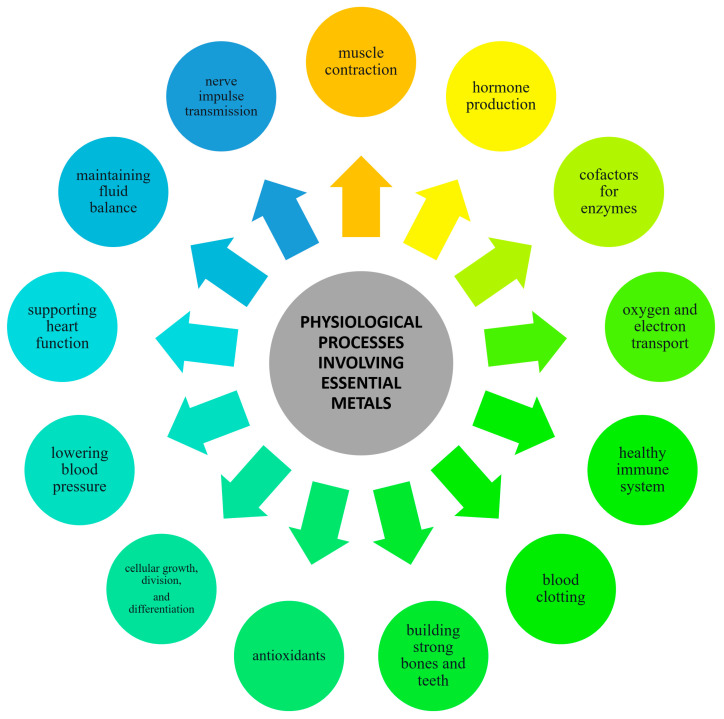
Physiological processes in which the essential metals are involved.

Metals present in our diets exert a wide range of effects on the human body. Essential metals like iron, zinc, and magnesium are integral to numerous biochemical reactions, including enzyme activity, oxygen transport, and cellular signaling. Without these metals, critical physiological processes would be significantly disrupted [6,7]. Trace metals, also known as trace elements or micronutrients, are essential metallic elements living organisms require in small quantities for proper physiological functioning. However, they become toxic to living organisms in excessive amounts [8]. Nevertheless, even essential metals can lead to health issues such as kidney damage, neuropsychiatric manifestations, and cardiovascular diseases if consumed excessively [9,10,11]. The balanced intake of metals through a varied diet is essential for these roles, contributing to structural functions, preventing diseases by supporting the immune system, facilitating growth, and regulating vital bodily functions such as heart rhythm and blood pressure. If the intake of these metals through the diet does not meet the lower limit for the proper function of the organism, supplements can be taken to compensate for their deficiency [12]. A scheme representing the physiological processes in which essential metals are involved is given in Figure 1.

**Figure 2:**
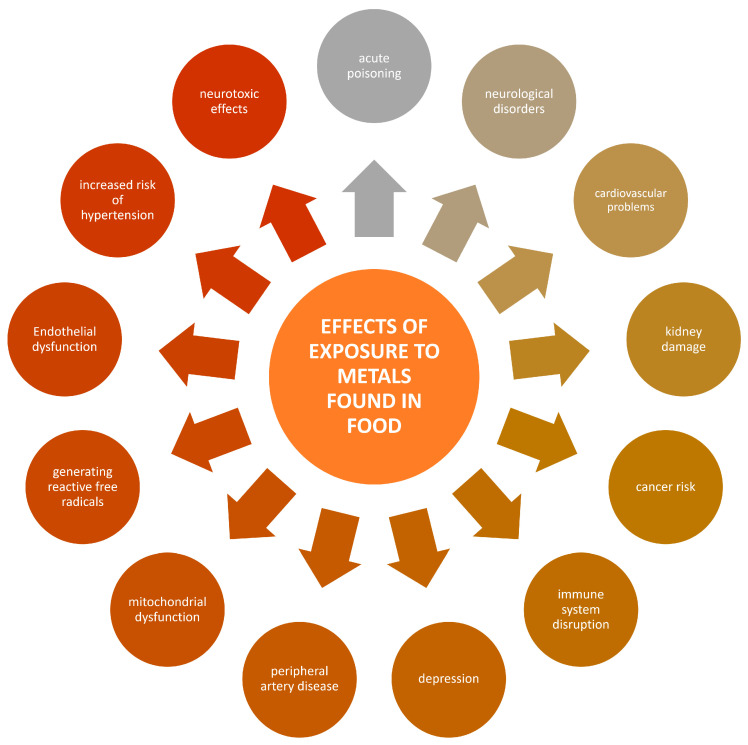
Potential threats resulting from food contamination with metals.

The ingestion of heavy metals such as lead, cadmium, and mercury poses severe health risks, including neurological damage, renal dysfunction, and elevated cancer risk, even at relatively low exposure levels [13,14,15]. According to World Health Organization (WHO) data, heavy metal ingestion represents a significant threat to human health [16]. Notably, food serves as the primary source of exposure for individuals who do not encounter these elements in their professional occupations. Naturally, food should be free from harmful metal contaminants to ensure safety and protect public health. Ingesting heavy metals through food can result in various health consequences, influenced by the type of metal, exposure level, and individual susceptibility [17]. Such exposure can lead to a spectrum of health effects, ranging from acute symptoms like nausea and organ failure [3] to chronic conditions such as neurological disorders like Alzheimer’s [18] and neurodevelopmental issues like attention deficit hyperactivity disorder (ADHD) and autism [19,20]. Heavy metals are also associated with significant kidney damage, potentially causing chronic kidney disease (CKD) and renal cancer [21]. They are implicated in an increased risk of various cancers by interfering with key transcription factors [22,23]. Moreover, heavy metals can impair the immune system, reducing cell function and increasing infection risks [24]. They also adversely affect the cardiovascular system, contributing to major cardiovascular diseases like hypertension and atherosclerosis, thereby increasing the risk of heart disease, stroke, and dementia [25,26,27,28,29,30,31]. These impacts are due to the metals inducing oxidative stress, inflammation, and endothelial dysfunction, among other mechanisms. A scheme representing potential threats resulting from food contamination with metals is given in Figure 2.

Metals in food originate from various sources, both natural and anthropogenic. Essential metals such as iron, zinc, copper, and magnesium naturally occur in the soil and are absorbed by plants, which we then consume directly or through animal products. However, industrial pollution, the use of fertilizers and pesticides, and contaminated irrigation water significantly contribute to the presence of toxic metals like lead, cadmium, and mercury in the food chain. These practices can lead to the accumulation of harmful metals in crops and livestock, thus posing potential health risks to humans. Combining these factors results in a complex scenario where food can simultaneously provide essential nutrients and carry toxic contaminants.

Considering that metals in food can be essential nutrients or harmful contaminants, this contribution aims to provide a comprehensive overview of the presence, significance, impact, and risks related to essential and toxic metals in our diet. It will discuss dietary recommendations for essential metals, address the risks associated with inappropriate intake levels, investigate the health implications of ingesting different metals, and explore regulations to limit the presence of non-essential metals in food. Our main goal is to comprehensively evaluate the dual role of food as both a source of beneficial elements and a potential vector for toxic metals. In particular, we aim to highlight how contemporary agricultural practices, industrial pollution, and the use of contaminated water and soil contribute to the presence of toxic metals in food. This review also seeks to underline the potential health risks associated with inadequate consumption of essential metals, which can become harmful in large quantities despite their necessity for human health. By analyzing current scientific data, we aim to provide a balanced perspective on the benefits and risks associated with the dietary intake of various metals.

## 2. Regulations and Standards Regarding the Presence of Heavy Metals in Food

Regulations governing the presence of heavy metals in foods are essential to protect public health and ensure the safety of consumable products. Heavy metals of major concern in these regulations involve lead, cadmium, mercury, and arsenic as a metalloid [3].

To analyze the quantity of heavy metals in food products, it is important to compare them with given reference values. Basic reference values define the maximum levels (ML) of specific metals in various types of food. Most recently, the European Commission published the Commission Regulation (EU) 2023/915 on maximum levels for specific contaminants in food and canceled Regulation (EC) No 1881/2006 [32]. The ML is established at a strict level that is reasonably attainable through good agricultural, fishery, and manufacturing practices, accounting for all possible health risks related to food consumption. Therefore, the ML levels for contaminants are set “as low as reasonably achievable” level. Based on this, food business operators can apply all possible measures and precautions for the prevention and/or reduction of food contamination to protect public health. All contaminants are listed in the Annex I of the Regulation (EU) 2023/915. This Regulation sets maximum lead, cadmium, mercury, and arsenic levels. Lead and cadmium levels were most recently updated in 2021, mercury levels in 2022, and arsenic levels in 2023. Heavy metal content is expressed as milligrams per kilogram (mg/kg) of the product’s wet weight; as stated in the Annex counsel, “The maximum level applies to the wet weight”.

Another regulation dealing with ML values is known under the name Codex Committee on Contaminants in Foods CF/11 INF/1, published by the Food and Agriculture Organization of the United Nations and World Health Organization in 2017 [33]. Besides the ML values for the safety factors of lead, cadmium, mercury, and arsenic, this regulation introduces the ML values for tin, copper, iron, and zinc as quality factors instead of safety factors. Furthermore, the Codex contains the toxicological guidance values expressed either as the provisional tolerable weekly intake (PTWI), the provisional tolerable monthly intake (PTMI), or the benchmark dose for a 0.5% increased incidence of lung cancer (BMDL 0.5). The PTWI and PTMI are expressed as micrograms per kilogram of body weight (µg/kg bw), and the BMDL 0.5 is expressed as micrograms per kilogram of body weight per day (µg/kg bw/day).

Wong et al. reviewed the daily maximum safe exposure levels for heavy metals [34]. This was accomplished by using data sourced from various regulatory bodies such as the United States Environmental Protection Agency (EPA), the Agency for Toxic Substances and Disease Registry (ATSDR), and the Joint FAO/WHO Expert Committee on Food Additives (JECFA). These agencies employ varying terms to denote exposure limits, including “Oral Reference Dose”, “Provisional Total Daily Intake”, or “Minimal Risk Level”. The collective term “reference values” was adopted to update the terminology and enhance clarity. All reference values integrated into the Heavy Metals Screening Tool (HMST) model are derived from dose–response data, which compares exposure levels with observed effects in either humans or laboratory animals. Toxicological advice considers the tolerable intake level of the contaminant for humans, expressed in micrograms (μg) per kg body weight (bw). Putting this into the framework, the estimated daily intake of the metal (μg/kg/day) was compared to both safe reference values and background exposure levels for a given metal. They focused on how updated default safe reference values and background exposure levels for arsenic, cadmium, lead, mercury, and chromium were chosen.

## 3. Essential Metals in Food

### 3.1. Sodium

Sodium is a crucial mineral for regulating body fluids and maintaining ion balance in the surrounding tissues through osmotic processes. It is the main cation in the extracellular fluid, significantly affecting the volume of fluids in the body. The kidneys manage sodium levels by filtering, reabsorbing, and secreting it within their operational units, the nephrons. Variations in sodium concentrations and blood pressure trigger the release of hormones by the kidneys that adjust water retention, blood pressure, and osmotic equilibrium [35]. It is also essential for the function of neurons and the transmission of nerve signals. It supports muscle contraction and activates certain enzymes. This mineral is key in creating the electrostatic potential across cell membranes, essential for conveying nerve impulses, with potassium as the complementary ion [36].

Most dietary sodium comes from table salt (NaCl), used for seasoning and preserving food, as it creates an osmotic gradient that prevents microbial growth. Iodized salt, essential for thyroid hormone production, is ideal for most cooking needs due to its quick dissolution and precise measurements, though its high sodium content (2360 mg of sodium per teaspoon) necessitates careful use [37]. Due to its large crystals, kosher salt contains less sodium per teaspoon (1240 mg) [38]. Low-sodium salt, beneficial for reducing sodium intake, replaces some sodium with potassium chloride, offering around 1770 mg of sodium per teaspoon, but requires caution for those with kidney disease [39]. Pink Himalayan salt, with trace minerals giving it a distinct color, offers a lower sodium alternative (1680 mg per teaspoon) and adds a unique flavor, though it lacks iodine [38]. Sea salt, derived from evaporated seawater, retains minor minerals and provides a strong flavor, with larger granules containing about 2000 mg of sodium per teaspoon [38] (Table 1).

Natural sources of sodium include milk (sodium content 50 mg/100 g), meats (sodium content 48 mg/100 g), and shellfish [40]. However, it is predominantly found in processed foods such as breads, crackers, processed meats, snacks (sodium content 1500 mg/100 g), and many condiments like soy (sodium content 7000 mg/100 g) and fish sauces [41]. As a result, diets rich in processed items and low in fresh produce tend to be high in sodium. The minimum sodium intake needed for optimal health is somewhat vague, but is thought to be between 200–500 mg per day [42]. Different health organizations have set varying guidelines for sodium reduction: the American Heart Association recommends less than 1500 mg/day [43], the WHO suggests less than 2000 mg/day [44], and the U.S. Department of Agriculture advises less than 2300 mg/day [44].

Sodium imbalance can lead to conditions like hyponatremia and hypernatremia, each with serious health implications [45]. Hyponatremia occurs when there is an abnormally low sodium concentration in the blood. Typically, sodium levels fall below 135 millimoles per liter (mmol/L). This condition often arises from excessive water intake, which dilutes the sodium in the body, or from conditions that increase water retention or sodium loss. Common causes include prolonged vomiting or diarrhea, heart failure, kidney disease, and excessive drinking of water. Symptoms of hyponatremia can vary based on the severity and rate of change in blood sodium levels, but they may include nausea, headaches, confusion, seizures, and, in severe cases, coma [46]. Treatment depends on the underlying cause but may involve fluid restrictions, salt tablets, or intravenous saline solutions to raise the sodium concentration carefully [47].

On the other hand, hypernatremia is characterized by a high sodium concentration in the blood, typically over 145 mmol/L. This usually results from dehydration—water loss without sufficient water intake—due to limited water access, excessive sweating, fever, or diseases such as diabetes insipidus that affect water reabsorption in the kidneys. Symptoms of hypernatremia include thirst, weakness, and confusion, and if the sodium levels rise too rapidly or too high, it can lead to seizures and coma as well. Managing hypernatremia involves treating dehydration with controlled water intake and monitoring the underlying condition that led to the sodium imbalance [48]. Both conditions are severe and can be life-threatening, requiring careful medical management to correct the sodium imbalance and address any complications or underlying conditions [47]. Extremely high doses of sodium, from 500 to 1000 mg/kg body weight, can cause acute symptoms like vomiting, gastrointestinal ulceration, and kidney damage. High intake is also linked to a heightened risk of developing kidney stones [49].

Furthermore, a systematic review and meta-analysis demonstrated that high dietary sodium significantly increases the risk of cardiovascular disease (CVD) by 19% compared to low sodium intake. A dose–response analysis revealed a linear relationship, with every 1 g increase in dietary sodium elevating CVD risk by 6% [50]. Research consistently shows that higher sodium intake correlates with elevated blood pressure, whereas reducing sodium intake can lower blood pressure in adults, whether they have hypertension or not. Several recent systematic reviews of randomized controlled trials have found that lowering sodium intake from usual or higher levels effectively reduces blood pressure [51]. Specific interventions aimed at changing behaviors to decrease sodium intake have successfully lowered blood pressure, both in individuals with and without hypertension. However, these reductions are modest, and the authors of these studies suggest that broader environmental strategies, such as reducing sodium content in processed foods, might lead to more significant decreases in sodium intake and, by extension, greater improvements in blood pressure levels [50,52].

Lowering sodium intake is crucial for patients with CKD to enhance the effectiveness of other treatments. Proteinuria, which indicates kidney disease and can accelerate its progression, is a key target for management. Elevated dietary sodium is associated with increased urinary albumin excretion and has been shown to diminish the benefits of angiotensin-converting enzyme (ACE) inhibitors, which are used to reduce proteinuria [11,53].

A recent meta-analysis established a direct link between dietary salt intake and an elevated risk of gastric cancer. The analysis shows that the risk increases progressively with higher levels of salt consumption. This finding is corroborated by both clinical and experimental studies, which suggest that high salt intake can affect the thickness of the gastric mucous barrier that protects the stomach lining. Additionally, excessive salt intake has been shown to enhance the colonization of Helicobacter pylori, a bacterium known to significantly increase the risk of developing gastric cancer [54,55,56].

### 3.2. Potassium

Potassium is an essential mineral and electrolyte vital for many physiological processes in the body. It helps maintain cellular function and overall health, making it indispensable for proper heart function, digestion, and muscle operations [57]. It is found naturally in a variety of foods. Rich sources include fruits such as bananas, oranges, and apricots; vegetables like cooked spinach, potatoes, and broccoli; legumes including lentils and kidney beans; and nuts and seeds like almonds and sunflower seeds. Whole grains, dairy products, and certain meats and fish like salmon and chicken can also be considered significant potassium sources [58,59,60]. An avocado offers about 507 mg of potassium per 100 g. Sweet potato contains about 229 mg of potassium for the same amount, while regular potato offers about 338 mg. Frozen spinach packs approximately 297 mg of potassium per 100 g, while raw spinach provides about 574 mg. Melon offers approximately 115 mg of potassium per 100 g. Coconut water is also a good source of potassium and delivers about 254 mg per 100 mL. White beans have about 552 mg of potassium per 100 g, while black beans value about 341 mg. Lentils contain approximately 356 mg of potassium per 100 g, and chickpeas provide about 237 mg. Soybeans contain approximately 451 mg of potassium per 100 g. Peanuts offer about 604 mg of potassium per 100 g [61].

One of the most important functions of potassium is its role in maintaining proper heart function. Potassium helps regulate the heartbeat by controlling the heart’s electrical activity, ensuring it beats regularly and efficiently. This is critical not only for heart health, but for the circulation of blood throughout the body. In addition to heart health, potassium is vital for muscle function, including voluntary muscles (like those used in walking and moving) and involuntary muscles (like the heart and digestive muscles). It helps to transmit signals that stimulate contractions necessary for muscle movement. This function is crucial for everyday activities and overall mobility [62]. Potassium also plays a significant role in nerve function by helping to transmit nerve signals that control various bodily functions, including movement, sensation, and cognition. This is essential for coordinating all bodily movements and reactions. Another significant benefit of potassium is its ability to counteract the effects of sodium in regulating blood pressure. High sodium levels can raise blood pressure, but potassium helps relax blood vessel walls and excretes excess sodium, thus aiding in maintaining healthy blood pressure levels. Finally, potassium is essential for bone health. It helps neutralize metabolic acids, which, if unchecked, can deplete the body’s calcium stores, leading to weakened bones. Therefore, adequate potassium intake is associated with improved bone health by helping to preserve calcium, which is crucial for maintaining strong bones. Potassium is integral to cardiovascular health, muscle and nerve function, fluid balance, and bone health, making it an essential nutrient for overall body maintenance and health [63].

Adults are recommended to consume about 3510 mg of potassium per day [64]. However, many adults do not meet these recommendations, which can negatively affect health. A potassium-rich diet has numerous health benefits, including a lower risk of cardiovascular diseases, kidney stones, osteoporosis, and reduced muscle wastage in older adults. Additionally, higher potassium intake is associated with a reduced incidence of stroke and lower overall mortality [65].

Numerous studies have demonstrated the health benefits associated with higher potassium intake. Both epidemiological and clinical research indicate that potassium-rich diets can lower blood pressure for people with hypertension and those with blood pressure within normal ranges [59,66]. Additionally, increasing potassium intake has been linked to reduced mortality from cardiovascular diseases, likely due to its effects on lowering blood pressure and possibly due to direct benefits on the cardiovascular system. High potassium diets may also help slow or prevent the progression of kidney disease and reduce urinary calcium levels, which helps manage hypercalciuria, reduce the risk of kidney stones, and decrease the likelihood of osteoporosis [67]. Furthermore, low potassium levels have been associated with an increased risk of glucose intolerance. Boosting potassium intake can help prevent diabetes that might develop from long-term use of thiazide diuretics. There is also evidence suggesting that low serum potassium increases the risk of deadly ventricular arrhythmias in patients with ischemic heart disease, heart failure, and left ventricular hypertrophy. Increasing dietary potassium, particularly through higher consumption of fruits and vegetables, is recommended to help mitigate these risks [68].

The two primary conditions associated with abnormal potassium levels are hyperkalemia (high potassium levels) and hypokalemia (low potassium levels). Hypokalemia occurs when there is an insufficient amount of potassium in the bloodstream. It can result from excessive potassium loss due to prolonged vomiting, diarrhea, excessive sweating, or the use of certain medications like diuretics and laxatives. It can also occur due to poor dietary intake, especially in people with eating disorders or those on extremely restricted diets. Conditions like renal tubular acidosis or Cushing’s syndrome can also lead to hypokalemia by interfering with potassium absorption or increasing potassium excretion. Symptoms of hypokalemia include muscle weakness, cramping, fatigue, and constipation. Severe hypokalemia can be life-threatening, leading to muscle paralysis, respiratory failure, or heart arrhythmias [69].

Hyperkalemia is characterized by abnormally high levels of potassium in the blood. It is usually caused by decreased potassium excretion due to kidney disease, certain medications such as ACE inhibitors, potassium-sparing diuretics, non-steroidal anti-inflammatory drugs, excessive potassium supplements, or potassium-rich foods, especially when combined with kidney issues or medications that reduce potassium excretion. Symptoms of hyperkalemia may include nausea, weakness, numbness, and slow heart rate. In severe cases, it can cause heart palpitations or sudden cardiac arrest [70]. Both hypo- and hyperkalemia can exacerbate chronic conditions or lead to long-term health issues if not appropriately managed. For example, CKD patients often struggle with potassium regulation, which can affect cardiovascular health and overall survival. Significant fluctuations in potassium levels can also impact blood pressure control, bone density, and muscle function, potentially contributing to falls and fractures in older people [71].

### 3.3. Magnesium

Magnesium is a vital mineral found abundantly in many foods, playing a crucial role in over 300 enzyme reactions in the human body. It is essential for the proper function of muscles and nerves, blood pressure regulation, and energy production [72]. The benefits of consuming adequate magnesium are significant. It helps maintain normal muscle and nerve function, keeps the heart rhythm steady, supports a healthy immune system, and keeps bones strong. Magnesium also helps regulate blood sugar levels, promotes normal blood pressure, and is known to be involved in energy metabolism and protein synthesis [73]. There is an association between higher dietary magnesium intake and reduced risk of conditions such as cardiovascular disease, hypertension, and diabetes [74]. Eating foods high in magnesium is also linked to a lower risk of osteoporosis and has been shown to improve mood and potentially reduce the risk of depression [75,76]. It has been found to mitigate the toxicity caused by cadmium. One potential mechanism is the enhancement of glutathione synthesis, which can counteract the toxic effects of cadmium [77]. Additionally, magnesium might compete with cadmium at the transporters responsible for its uptake, decreasing its absorption. As a result, magnesium’s presence can reduce lipid peroxidation and oxidative stress. This protective effect is likely because magnesium is a cofactor for essential enzymes in reducing the reactive oxygen species (ROS) [21,78].

Rich dietary sources of magnesium include leafy green vegetables like spinach, nuts such as almonds and cashews, seeds, whole grains, and legumes. Beans, bananas, dried fruit, and dark chocolate are also good sources of this mineral [79]. The daily recommended value for magnesium is 420 mg for adults and children aged 4 years and older, according to the U.S. Food and Drug Administration (FDA) [80]. Pumpkin seeds provide approximately 557 mg of magnesium per 100 g. Chia seeds offer around 396 mg of magnesium per 100 g. Almonds, when dry-roasted, contain roughly 286 mg of magnesium per 100 g. Boiled spinach provides about 78 mg of magnesium per 100 g. Cashews, dry-roasted, offer approximately 264 mg of magnesium per 100 g, while oil-roasted peanuts contain about 175 mg for the same amount. A baked potato with skin provides around 43 mg of magnesium per 100 g. Cooked brown rice contains approximately 42 mg of magnesium per 100 g, and plain, low-fat yogurt provides around 19 mg. Raisins contain about 23 mg of magnesium per 100 g, while cooked broccoli contains around 12 mg, and cooked white rice has about 10 mg. Roasted chicken breast provides approximately 26 mg per 100 g, while pan-broiled, 90% lean ground beef offers about 23 mg. An apple provides around 9 mg, and a raw carrot contains approximately 7 mg per 100 g [39].

However, while magnesium is essential for health, its excessive intake via food supplements can lead to side effects. These include diarrhea, which can occur when the body excretes excess magnesium to balance the levels. Hypermagnesemia is a rare but severe condition that occurs when excessive magnesium is in the blood [81]. This condition is most commonly caused by excessive magnesium intake through supplements or medications rather than from dietary sources because the body can naturally regulate the levels of magnesium derived from food [82]. The kidneys typically filter out excess magnesium efficiently; however, magnesium toxicity can occur primarily in individuals with renal impairment or kidney failure, where the kidneys lose the ability to remove surplus magnesium. It can also arise from excessive intake of magnesium-containing laxatives and antacids [83].

Low magnesium levels, or hypomagnesemia, can significantly impact the body due to magnesium’s role in enzymatic reactions and various physiological processes. Early symptoms of magnesium deficiency are often mild or non-specific, making it easy to overlook until they become more severe [84]. Common causes of low magnesium include poor dietary intake, where individuals do not consume enough magnesium-rich foods like green leafy vegetables, nuts, and whole grains. Increased losses through excessive urination or diarrhea, chronic health conditions such as diabetes, gastrointestinal diseases, and alcohol abuse also contribute to lower magnesium levels. Medications like diuretics, antibiotics, and chemotherapy agents can also increase magnesium excretion. Symptoms of low magnesium can range from neuromuscular issues such as muscle weakness, tremors, atherosclerosis, cramps, and spasms to severe cases leading to convulsions [85]. It also affects cardiovascular health, potentially leading to irregular heart rhythms (arrhythmias) that increase the risk of heart attacks. Mental health is also impacted, with common symptoms including irritability, anxiety, lethargy, fatigue, and, in severe cases, depression and confusion. Other effects of magnesium deficiency can include nausea, loss of appetite, and vomiting. Over the long term, low magnesium can weaken bones; increase the risk of osteoporosis; and contribute to the development of high blood pressure, heart disease, and type 2 diabetes [83,86].

### 3.4. Calcium

Calcium is an essential mineral in the human body, and it has several biological implications that affect various physiological processes. It is vital for developing and maintaining strong bones and teeth, as it forms part of the structural framework of the skeleton. Beyond its role in bone health, calcium is essential for muscle function, allowing muscles to contract and relax properly. This is particularly important for the heart muscle, where calcium regulates the beating and rhythm [87]. Calcium also plays a critical role in nerve transmission, acting as a messenger between nerve cells and between nerves and muscles, facilitating the propagation of electrical impulses throughout the nervous system. This is crucial for all motor and sensory functions. Adequate calcium intake is essential for preventing osteoporosis and bone fractures, particularly as people age [88]. Furthermore, calcium serves as a signaling molecule within cells [89]. It helps regulate cellular processes, such as cell division and gene expression, by acting as a secondary messenger in many hormone and neurotransmitter pathways. This regulation is critical for maintaining the health and function of cells and for the body’s response to external signals [90].

Calcium is abundant in food sources such as dairy products, leafy greens, and fortified foods [91]. The recommended daily allowances for calcium are 200–260 mg for children (0–12 months), 700–1300 mg for children (1–18 years), and 1000–1300 mg for adults (older than 18) [92]. Different types of milk vary in their calcium content. Semi-skimmed milk contains around 240 mg of calcium per 200 mL serving, while skimmed milk offers slightly more at 244 mg. Whole milk contains approximately 236 mg of calcium in the same serving size. Milkshakes are a richer source of calcium, providing about 360 mg per 200 mL. Sheep milk contains even more calcium, offering around 380 mg. On the other hand, coconut milk has a lower calcium content, with only about 54 mg per 200 mL serving. Non-enriched soy drinks contain around 26 mg, whereas calcium-enriched soy drinks match the calcium content of semi-skimmed milk at approximately 240 mg. Rice drinks and oat milk provide only minimal amounts of calcium, with approximately 22 mg and 16 mg, respectively, per 200 mL serving. Almond milk contains about 90 mg of calcium in the same serving size [93] (Table 2).

Excessive calcium intake can lead to several health problems, particularly when it results in hypercalcemia or high calcium levels in the blood. This condition can cause various symptoms and complications that affect various body systems. One of the immediate effects of hypercalcemia is the disruption of normal kidney function. High calcium levels can lead to the formation of kidney stones. Over time, persistent hypercalcemia can contribute to kidney damage and decreased kidney function [94]. In the digestive system, excessive calcium can cause constipation and, in some cases, might contribute to the development of stomach ulcers or gallstones [95]. Neurologically, high calcium levels can result in fatigue, lethargy, and, in severe cases, confusion or even coma [96,97]. It can also affect the heart, potentially leading to arrhythmias or other cardiovascular issues [98]. Chronically high calcium levels may also interfere with the absorption of other minerals, such as iron and zinc, potentially leading to deficiencies in these essential nutrients. Over-supplementation, excessive dietary intake, or underlying medical conditions such as hyperparathyroidism, which involves increased secretion of parathyroid hormone, raises calcium levels [99].

Low calcium levels in the body, known as hypocalcemia, can lead to several health issues affecting the bones, muscles, and nervous system [100]. Chronic low calcium can cause osteoporosis, where bones become fragile and are more likely to break. A less severe form, osteopenia, involves lower than normal bone mineral density and can be a precursor to osteoporosis [101]. Children with prolonged low calcium intake can develop rickets, which results in skeletal deformities such as bowed legs [102]. In adults, the same deficiency leads to osteomalacia, characterized by bone pain and muscle weakness. Extremely low calcium levels can cause tetany, which includes muscle spasms and tingling in the lips, hands, and feet. Severe cases can lead to convulsions or tetanic seizures [103]. Hypoparathyroidism, where the body produces insufficient parathyroid hormone crucial for calcium regulation, also results from low calcium. This can cause muscle cramps and tingling in the fingers [104].

Additionally, inadequate calcium can weaken teeth, increasing decay and susceptibility to periodontal diseases. Some evidence suggests that low calcium levels might increase the risk of developing cataracts [105]. Causes of low calcium levels include dietary deficiencies, hormonal changes, certain medications, and genetic factors [106]. Treatment often involves addressing the underlying cause, including calcium supplementation or dietary adjustments to ensure adequate intake.

### 3.5. Zinc

Zinc is an essential mineral crucial in numerous aspects of cellular metabolism. It acts as a catalyst for more than 300 enzymes involved in synthesizing and metabolizing carbohydrates, lipids, proteins, and nucleic acids. Zinc is essential for the functioning of the immune system, wound healing, DNA synthesis, and cell division. It is vital for proper growth and development during pregnancy, childhood, and adolescence [107]. Zinc is required to treat skin conditions, including infections, acne, and ulcers [108]. Zinc deficiency may increase the risk of infections; it helps activate T cells, which control and regulate the immune response [109]. The skin contains approximately 5% of the body’s total zinc content, which plays a role in cell growth, collagen formation, and inflammatory responses, making it essential for proper wound healing [110].

Research has demonstrated that zinc can reduce kidney toxicity triggered by cadmium, potentially by inhibiting cadmium’s ability to disrupt antioxidant enzymes. Additionally, zinc promotes the expression of metallothionein in the liver and kidneys. The increased MT expression aids in forming MT-Cd complexes in these organs, which helps retain cadmium in a non-toxic form. Furthermore, zinc decreases cadmium-induced cell death (apoptosis) in kidney cells and may compete with cadmium to enter cells through specific transporters [21].

Zinc is found in a wide range of foods. High zinc concentrations are present in red meat, poultry, and seafood such as oysters and crab. Dairy products like cheese and milk also provide zinc. Plant-based sources include beans, nuts, whole grains, and fortified cereals. However, the bioavailability of zinc from plant sources is generally lower than from animal sources due to the presence of phytates, which inhibit zinc absorption [111]. Oysters are particularly rich in zinc, containing approximately 33 mg per 100 g (depending on the variety). Beef steak provides 4.2 mg of zinc per 100 g, while pumpkin seeds offer 7.3 mg. Crab contributes 3.6 mg of zinc per 100 g, while oats cooked in water contain 2.6 mg. Cheddar cheese provides 3.3 mg of zinc per 100 g, and canned sardines drained of oil offer 1.2 mg per 100 g. Milk with 1% fat content contains 1.1 mg of zinc per 100 g, while peanuts offer 2.7 mg. An egg provides 2.7 mg of zinc per 100 g, and cooked salmon contributes 0.6 mg per 100 g. White rice offers 0.7 mg of zinc, and white bread contains 0.5 mg per 100 g [112].

The body has no specialized zinc storage system, so regular consumption of zinc-rich foods is important to maintain a steady state of this essential mineral. The ML values for zinc are still not established, while the PMTDI is 0.3–1 mg/kg bw [33]. The recommended daily allowance for zinc varies by age, sex, and life stage, but typically, adults require between 8 and 11 milligrams per day [113].

Zinc bioavailability tends to be lower in vegetarian diets compared to non-vegetarian diets due to the high consumption of legumes and whole grains among vegetarians. These foods contain phytates, substances that bind with zinc and inhibit its absorption. Additionally, meat, absent in vegetarian and vegan diets, is a rich source of bioavailable zinc. Consequently, vegetarians and vegans often have lower dietary zinc intakes and reduced serum zinc levels than meat consumers [114].

Zinc deficiency can lead to various health issues, including impaired immune function, slowed growth in children, hair loss, diarrhea, and delayed wound healing [115]. On the other hand, excessive zinc intake, usually from supplements, can cause toxicity symptoms such as nausea, vomiting, loss of appetite, stomach cramps, diarrhea, and headaches. Long-term high zinc intake can interfere with the body’s absorption of other essential minerals, such as copper and iron [116].

### 3.6. Iron

Iron is a vital mineral essential for various bodily functions, most notably for the formation of hemoglobin, the protein in red blood cells that carries oxygen to the body’s tissues. Iron is also necessary for the proper function many enzymes and the production of certain hormones [117,118]. Two types of iron are found in foods: heme and non-heme iron. Heme iron is derived from hemoglobin and is found in animal products such as red meat, poultry, and fish. The body more readily absorbs it compared to non-heme iron. Non-heme iron, which makes up most of the iron in diets, is found in animal and plant sources like lentils, beans, tofu, cooked spinach, fortified cereals, and whole grains [119].

Iron-rich foods can be categorized into heme iron and non-heme iron sources. Heme iron, found in animal products, includes liver (pork, chicken, or beef), with 6.13 to 17.87 mg per 100 g; oysters or mussels, with 6.67 to 8.40 mg per 100 g; lamb or beef, with 2.0 to 3.2 mg; clams, with 2.8 mg; sardines, with 2.7 mg per 100 g; tuna, herring, trout, or mackerel, with 1.6 mg per 100 g; chicken or pork, with 1.2 mg; and salmon or turkey, with 0.7 mg per 100 g. Non-heme iron sources, prevalent in plant-based foods, comprise soybeans, with 3.7 mg per 100 mL; beans or lentils, with 1.9 to 2.8 mg; and roasted pumpkin seeds/kernels, with 7.8 mg per 100 g [120].

The absorption of iron from food depends on various factors. For instance, vitamin C significantly enhances the absorption of non-heme iron when consumed in the same meal. In contrast, substances like phytates, found in some grains and legumes; calcium; and certain polyphenols in tea and coffee can inhibit it [121]. Iron testing mainly targets oils and fats. The regulated iron content in these products varies from 1.5 to 7 mg/kg, with the PMTDI being 0.8 mg/kg bw [33].

The recommended daily iron intake varies depending on age, gender, and life stage, such as pregnancy. For example, adult men and postmenopausal women generally need about 8 mg daily, whereas premenopausal women require about 18 mg daily due to iron loss during menstruation. The tolerable upper intake limits for iron have been set to 40 mg per day for children up to 13 years and to 45 mg per day for older individuals [122].

Iron deficiency is one of the most common nutritional deficiencies worldwide, leading to anemia, characterized by symptoms such as fatigue, weakness, and pale skin [123]. However, excessive iron intake can lead to toxicity and cause serious health problems, including liver damage and heart problems [124]. The World Health Organization has noted that in 2019, about 30% of women of childbearing age worldwide were affected by anemia, as well as about 40% of children [125]. One of the primary strategies for preventing iron deficiency is dietary diversification, which involves creating balanced meals to meet iron needs that can be easily implemented and accepted. However, the effectiveness of consuming a diversified diet is often constrained by economic, social, and cultural factors [126]. For instance, increasing heme iron intake from animal sources can significantly diminish the risk of anemia. Still, excessive consumption of animal products might lead to hypertension, type 2 diabetes, chronic diseases, and gastrointestinal cancers [127]. As such, careful consideration is necessary when implementing iron supplementation.

While iron deficiency remains a concern globally, there is growing awareness that iron overload could be a more significant issue in Western countries. This is attributed to the fortification of cereals, the widespread use of iron supplements, and the high consumption of red meat. Iron overload is an excess accumulation of iron in the body, mainly stored in ferritin and hemosiderin. Research indicates that about 10% of postmenopausal women in these regions exhibit elevated ferritin levels, suggesting a prevalent issue of iron overload [128]. Iron, a transition metal, possesses loosely bound electrons in its outer shell, which can catalyze the production of ROS, thereby increasing oxidative stress. This stress involves mutations, DNA single- and double-strand breaks, and activation of oncogenes. Oxidative stress occurs when there is a disturbance in the balance between ROS production and the body’s antioxidant defenses, leading to excess ROS. These ROS, or free radicals, contain an unpaired electron, making them unstable and reactive with DNA and other cellular molecules [129].

Epidemiological studies that employ validated biomarkers to measure body iron stores and iron intake are necessary to further understand the relationship between iron levels and cancer risk [130]. Iron may also interact with other factors in breast carcinogenesis [131]. For instance, the metabolism of estradiol to catechol estrogen, which forms semi-quinones and quinones, is a crucial step in the process. Iron can exacerbate estrogen-induced carcinogenesis through several mechanisms, such as replacing zinc in the DNA-binding domain of the estrogen receptor. In this altered state, known as the “iron finger”, the receptor can produce free radicals in hydrogen peroxide and ascorbate [132,133].

Moreover, estrogen administration has been shown to increase iron accumulation in animal models and enhance iron uptake in cell cultures. This process is complemented by the metabolic cycling of estrogen metabolites, which generates superoxide radicals that release iron from ferritin storage [134]. Animal studies have shown that a combination of estradiol and an iron-rich diet significantly increases the incidence and number of kidney tumor nodules compared to a low-iron diet [135].

### 3.7. Copper

Copper is an essential trace mineral that is vital in various bodily functions. It is necessary for red blood cell production and maintenance of the nerve cells and the immune system. Copper also helps the body form collagen, a key part of bones and connective tissue, and is involved in energy production at the cellular level [136]. The body needs copper to effectively utilize iron and carry out several important enzymatic reactions that contribute to energy production. Copper also acts as an antioxidant, helping to protect cells against the damage induced by the overproduction of free radicals [137].

Typically, people consume enough copper in the foods they eat, as it is found in a wide range of foods, which makes copper deficiency relatively rare in most populations with a balanced diet. The richest dietary sources of copper include shellfish, seeds and nuts, whole-grain products, wheat bran cereals, organ meats such as liver, chocolate, and some fruits and vegetables. Drinking water through copper pipes can also contribute to dietary intake [138,139]. The ML values for copper are established for specific food categories, such as milk fat products and oils, ranging from 0.05 to 0.4 mg/kg; salt, 2 mg/kg; and casein products, 5 mg/kg. PMTDI toxicological guidance values range between 0.05–0.5 mg/kg bw [33]. Disruption of copper homeostasis may lead to serious medical conditions, such as Menkes disease (kinky hair syndrome), a rare copper metabolism disorder. It occurs in male infants and affects their copper levels [140].

The copper content varies significantly in different food categories. Beef liver contains approximately 14.59 mg of copper per 100 g, while oysters offer around 5.71 mg. Unsweetened baking chocolate contains approximately 3.35 mg per 100 g, and cooked potatoes, flesh, and skin provide about 0.49 mg per 100 g. Shiitake mushrooms contain around 1.35 mg per 100 g, and dry-roasted cashew nuts offer approximately 2.24 mg. Dungeness crab provides about 0.73 mg per 100 g, while toasted sunflower seed kernels contain approximately 2.05 mg per 100 g. Turkey giblets offer around 0.69 mg per 100 g, and dark chocolate (70–85% cacao solids) contains approximately 1.79 mg per 100 g. Raw tofu provides about 0.4 mg, and cooked chickpeas offer around 0.24 mg per 100 g. Whole wheat pasta provides approximately 0.15 mg per 100 g, and raw avocado contains around 0.18 mg. Dried figs offer approximately 0.29 mg per 100 g, while boiled spinach contains about 0.18 mg per 100 g. Cooked asparagus provides approximately 0.17 mg per 100 g, and sesame seeds offer around 0.49 mg per 100 g. Turkey meat contains approximately 0.15 mg per 100 g, tomatoes contain approximately 0.04 mg, while plain low-fat Greek yogurt offers around 0.02 mg per 100 g. Nonfat milk provides about 0.01 mg per 100 g, and raw apples with skin contain approximately 0.02 mg per 100 g [39].

Copper deficiency is linked to changes in blood lipid levels, which can elevate the risk of atherosclerotic cardiovascular disease [141]. Animal studies have shown that insufficient copper can cause heart abnormalities due to decreased activity in cardiac cuproenzymes [142]. There is also speculation that low dietary copper could play a role in the onset and progression of Alzheimer’s disease, the leading cause of dementia. This hypothesis is supported by observations of lower copper levels and diminished activity of copper-dependent enzymes in the brains of Alzheimer’s patients. Conversely, some studies suggest that higher copper levels might reduce the risk of Alzheimer’s. Still, others have found increased copper levels in the brains of those with the disease, implying that excessive copper intake might contribute to its development. Moreover, copper accumulation in the brain regions affected by Alzheimer’s does not necessarily correlate with the overall body’s copper status or intake [143].

While copper deficiency can lead to health problems, excess copper can also be toxic. The body has a natural mechanism to regulate copper absorption and excretion to prevent these issues. Still, in cases of genetic disorders such as Wilson’s disease, this regulation is impaired, leading to copper accumulation and associated health risks [144]. Wilson’s disease causes deposits of copper in the liver, brain, and other organs. The increased copper in these tissues leads to hepatitis, kidney compromise, brain disorders, and other problems [145].

Long-term exposure to high levels of copper can lead to liver damage and gastrointestinal problems, including abdominal pain, cramps, nausea, diarrhea, and vomiting [146]. While copper toxicity is rare in individuals without genetic conditions affecting copper metabolism, there have been cases of copper toxicity from drinking water with high copper levels, often due to stagnation in copper pipes and fixtures or leaching from copper alloys in water distribution systems and household plumbing [139]. The Environmental Protection Agency has set a recommended copper limit of 1.3 mg/L in public water systems to help prevent these health risks [147].

### 3.8. Cobalt

Cobalt is a trace mineral that plays a crucial role in the human diet, mainly because it is a part of vitamin B12, which is crucial for the health of nerve and blood cells and DNA synthesis [148]. Vitamin B12 is unique among vitamins as it includes a metal ion. It acts as a cofactor for critical enzymes such as methionine synthase and methyl malonyl-CoA mutase, which are involved in synthesizing and breaking amino acids and fatty acids in bacteria and mammals [149]. This makes it essential for maintaining bodily equilibrium. In humans, a cobalamin deficiency, in the form of vitamin B12, can reduce the activity of these enzymes, leading to megaloblastic anemia [150]. The inclusion of cobalt in vitamin B12 aids in maintaining nerve cell health, forming red blood cells, and preventing megaloblastic anemia, characterized by fatigue and weakness [151]. B12 is essential for cellular metabolism, affecting DNA regulation, synthesis, and energy production.

Cobalt is found in foods that are rich in vitamin B12. Animal products, taken up with the diet, are humans’ only natural source of vitamin B12. These include animal liver and kidney; fish and shellfish; meats like beef, lamb, and pork; and dairy products like milk, cheese, yogurt, and eggs [152]. For those who do not consume animal products, fortified foods like plant-based milk alternatives, breakfast cereals, and nutritional yeasts can provide the necessary vitamin B12. Vegans can be at risk of dietary deficiency of vitamin B12 because their diet, based only on plant foods (vegetables, grains, nuts, and fruits), does not contain this vitamin. Vegans do not eat foods that come from animals, including dairy products and eggs. Vegan diets have to include fortified foods and supplements containing cyanocobalamin (the synthetic form of vitamin B12), vitamin D, selenium, iodine, calcium, iron, and nutritional yeast [153,154]. The average adult intake of cobalt is 5 to 60 µg per day [155]. A safe recommended dietary allowance for cobalt has not yet been set.

The cobalt content in various food categories varies significantly. Cereals and cereal products exhibit a range from 4.03 to 13.11 µg/kg, while meat and meat products contain between 2.01 and 5.52 µg/kg. Milk and dairy products show a wider range, from 3.60 to 12.24 µg/kg, whereas eggs range from 0.94 to 5.48 µg/kg. Fish and seafood offer cobalt concentrations between 2.98 and 7.45 µg/kg. Vegetables encompass a broader range, from 3.71 to 13.92 µg/kg, whereas legumes exhibit significantly higher levels, varying from 39.28 to 104.29 µg/kg. Potatoes contain between 5.07 and 12.80 µg/kg of cobalt, while fresh fruits range from 1.97 to 6.43 µg/kg. Dry fruits, nuts, and seeds show a wider spectrum, with concentrations spanning from 16.64 to 89.51 µg/kg. Sweets, chocolate, cakes, and similar products exhibit cobalt levels ranging from 3.32 to 69.53 µg/kg. Oils and fats have the lowest cobalt content, ranging from 0.01 to 1.77 µg/kg, and beverages range from 0.52 to 3.06 µg/kg [156].

Exposure to metallic cobalt, especially in industrial settings, can be harmful, causing issues like cardiomyopathy, thyroid damage, and respiratory complications. However, these are typically related to occupational exposure rather than dietary intake. Food-derived cobalt toxicity is rare [157].

Furthermore, low vitamin B12 intake can lead to serious health concerns such as fatigue, various types of anemia, and potential kidney and liver damage, as well as neurotoxic effects [158,159]. Vitamin B12 deficiency can cause severe anemia and other health complications. It is especially crucial during pregnancy, with recommended intakes rising significantly. Vitamin B12 plays a significant role in immune system regulation. Deficiencies in B12 and folic acid can alter immune responses and have been linked to an increased risk of diseases like Alzheimer’s and systemic inflammation [160]. Studies have also pointed to B12’s potential in treating viral infections and its effects on conditions like ischemic stroke in the elderly [161]. Those at the highest risk include vegans, pregnant women, infants of deficient mothers, and type 2 diabetes patients on metformin [162].

On the other hand, high levels of cobalt exposure can result in eye issues, thyroid and heart damage, diminished lung function, tinnitus, hearing loss, cardiomyopathy, and hypothyroidism, highlighting the health challenges related to both deficiency and excessive exposure to cobalt and vitamin B12 [163,164]. It can be harmful to the heart muscle, potentially leading to heart muscle disease (toxic cardiomyopathy) when one is exposed to excessive amounts. Elevated levels of cobalt can also result in an increased production of red blood cells (polycythemia), which, if unaddressed, might lead to congestive heart failure [165]. Excessive consumption of cobalt can cause the thyroid gland to enlarge (goiter) and decrease thyroid function [166].

### 3.9. Chromium

Non-occupational human populations are exposed to chromium primarily by consuming food and water containing chromium or through dermal contact with its products [167]. Chromium has multiple oxidation states, of which the trivalent Cr(III) and hexavalent Cr(VI) forms are the most common biologically and environmentally stable forms [168]. After absorption, Cr(VI) is converted to Cr(III) and excreted in urine and feces. Ingestion of Cr(VI) can result in acute renal failure and tubular necrosis [169]. Cr(III) possesses low membrane permeability and cannot penetrate the cell membrane, which confines it within the cell where it can bind to DNA, causing genetic damage and genomic instability [167]. Also, it can induce cell membrane lipid injuries, disturbing cell function and integrity [170]. While Cr(III) is considered an essential nutrient and may help maintain normal glucose tolerance, there are safety concerns regarding its supplemental intake due to increasing evidence of genotoxicity [171]. Despite these concerns, the scientific consensus supports its use as long as the daily intake does not exceed 250 μg/d [172]. The mechanism behind chromium-induced kidney damage is not well understood. In rats, chromium can accumulate in the renal cortex at much higher levels than in other tissues. Studies in animals have shown that chromium can cause cell damage, DNA damage, and oxidative stress in the kidneys, suggesting a potential for nephrotoxicity in humans [173].

The chromium content in different food items varies widely. Mussels contain the highest concentration, with 128 µg per 100 g, followed closely by Brazil nuts at 100 µg. Oysters offer around 57 µg, while dried dates and pears contain 29 µg and 27 µg, respectively. Brown shrimp and wholemeal flour provide 26 µg and 21 µg, respectively. Tomatoes offer approximately 20 µg, while mushrooms contain 17 µg of chromium. Broccoli provides 16 µg, while wholegrain barley contains 13 µg. Hazelnuts offer around 12 µg, and pork chops provide 10 µg. Wholegrain maize contains 9 µg, while egg yolks offer 6 µg. Beef and herring have lower chromium content, with 3 µg and 2 µg, respectively, per 100 g [174].

Numerous studies have suggested an association between chromium exposure (particularly Cr(VI)) and cancer. Cr(VI) compounds can penetrate cells through sulfate-anion channels and undergo reduction by glutathione and ascorbate, which leads to the formation of highly reactive Cr(V/IV) intermediates, finally resulting in Cr(III) products [175]. Excessive ROS production during these processes can lead to oxidative stress and DNA damage, such as chromium-DNA adducts, DNA strand breaks, DNA inter/intrastrand cross-links, DNA–protein cross-links, and p53 point mutations [176]. Due to these properties, the International Agency for Research on Cancer (IARC) has classified Cr(VI) as a Group I carcinogen, indicating its potential to cause cancer in humans [177]. Exposure to chromium has been linked to the development of several cancers, including the lungs, larynx, bladder, kidneys, stomach, testicles, bone, and thyroid cancer [168,170].

Exposure to chromium can also lead to various immune reactions, which depend on the dose, the route of exposure, and the type of chromium compound. Typically, inhaling particulate chromium compounds causes lung damage and triggers a significant lung inflammatory response [178]. Cr(III) is essential for normal glucose metabolism, and inadequate dietary intake has been associated with cardiovascular disease and diabetes [179]. Numerous studies have shown that Cr(III) can act as an immune modulator either by immunostimulatory or immunosuppressive mechanisms through its effect on B cells, T cells, and macrophages, as well as on cytokine production [180]. Considering the effects of Cr(III) supplementation on inflammatory mediators in human populations, a recent systematic review and meta-analysis of randomized controlled trials has shown that chromium picolinate and chromium chloride at dosages below 300 μg for up to 12 weeks may decrease the serum level of C-reactive protein. Chromium picolinate and chromium dinicocysteinate did not affect the serum levels of IL-6 and IL-8, while the level of TNF-α was reduced by chromium dinicocysteinate treatment but not by chromium picolinate [181]. Future research should focus on establishing a standard for measuring chromium status to identify populations with deficiency and reevaluate the metabolic response to chromium supplementation. Additional randomized, double-blind controlled trials are required in order to achieve consistent findings and make relevant clinical recommendations.

## 4. Toxic Metals in Food

### 4.1. Lead

Lead is a potent neurotoxin, and the main sources of dietary lead are fish (0.002–0.161 mg/kg), cereal products, grains, vegetables (especially potatoes and leafy greens), and tap water. The ML values for lead range from 0.01 mg/kg in the case of some foods placed on the market for infants and young children to 3 mg/kg for some food supplements. Meat products, fats, and oils are typically restricted to 0.1 mg/kg of lead, while fruit, vegetables, and fungi have limits ranging from 0.1 mg/kg to 0.8 mg/kg. The limits for fruits, vegetables, and fungi vary between 0.05 mg/kg and 0.8 mg/kg. For wine, cider, perry, and fruit wine, limits vary between 0.1 mg/kg and 0.2 mg/kg, with the tendency to be less than 0.1 mg/kg for products made from the 2022 fruit harvest. The toxicological guidance value PTWI is withdrawn [32,33].

The central nervous system (CNS) is particularly vulnerable to lead toxicity, with the developing brain being more susceptible than the mature one. Significantly, epidemiological research consistently links lead exposure to cognitive deficits, behavioral problems, and neurodevelopmental disorders in children [182]. Studies have shown that prenatal and early-life exposure to lead is associated with a range of adverse outcomes, including reduced IQ, deficits in motor function, attention deficits, hand-eye coordination issues, and learning disabilities later in life [183,184,185]. Moreover, data from seven international longitudinal cohorts involving 1333 children, performed in 2003, revealed an inverse relationship between blood lead concentration and IQ scores, indicating that even low levels of lead, below the allowed level (7.5 μg/dL), can significantly disrupt cognitive development and cause intellectual deficits in children [184]. A prospective study by Bellinger and colleagues found that infants (from birth to two years) with high prenatal lead exposure exhibited lower developmental scores compared to those with low or medium exposure levels [183].

In adults, chronic and extreme lead exposure can cause multiple neurological disorders, such as cataracts, nerve disorders, memory or concentration problems, lack of muscular coordination, etc. [182]. A neuroimaging study (Cincinnati Lead Study) using magnetic resonance imaging (MRI) and functional MRI (fMRI) performed in 157 adults detected structural and functional alterations in brain morphology in individuals with a history of lead exposure [186]. These changes included reductions in gray matter volume in the prefrontal lobe and abnormalities in white matter integrity, followed by disrupted neural circuitry underlying cognitive impairments [186].

Animal research has shown that lead exposure during the prenatal and early postnatal stages can lead to long-lasting alterations in brain structure and function, supporting human data [187]. Lead disrupts various cellular processes within the CNS, particularly in the hippocampus, affecting glutamatergic and cholinergic systems [188]. The major molecular mechanism relies on reversible inhibition of the NMDA and AMPA-activated calcium channel, thus preventing the influx of Ca^2+^ into the postsynaptic neuron and leading to impaired synaptic transmission and altered neuronal excitability [189]. An electrophysiological study done by Wang and colleagues performed in juvenile rats has confirmed that lead exposure blocks NMDA and AMPA receptors alter long-term potentiation (LTP) induction and causes a significant decline in the density of dendritic spines in the CA1 part of the hippocampus [190]. Animal studies have also revealed that lead exposure can induce oxidative stress, inflammation, and apoptosis in the brain, further contributing to neuronal damage and cognitive impairments [168]. The main target for the accumulation of lead within cells is mitochondria, and its overload can induce oxidative stress and depletion of mitochondria-generated ATP energy metabolism, which further contributes to the disruption of neuronal function [191,192]. Moreover, research conducted in non-human primates has provided evidence of lead-induced alterations in brain development and behavior, highlighting the translational relevance of findings from animal models to humans.

Chronic lead poisoning may result in lead nephropathy, characterized by kidney damage such as tubulointerstitial fibrosis, tubular atrophy, and glomerular sclerosis, resulting in a reduced glomerular filtration rate (GFR) [193]. Even low-level lead exposure, indicated by blood lead levels below 10 µg/dL, has been linked to an increased risk of CKD, particularly in individuals with hypertension or diabetes [193]. In a study of North American children with CKD, higher blood lead levels were associated with a decrease in GFR, especially in those with glomerular disease underlying CKD [194]. Lead induces ROS production and oxidative stress, causing lipid peroxidation and cellular damage. Accumulation in mitochondria leads to structural and functional changes, including swelling and inhibition of respiratory function and ATP production, impairing energy-dependent processes such as tubular transport. Lead also inhibits mitochondrial enzymes such as aminolevulinic acid synthase and ferrochelatase and affects a heme-containing hydroxylase enzyme responsible for converting 25-hydroxy vitamin D into 1,25-dihydroxy vitamin D [195,196]. Even a small amount of absorbed lead can cause cellular malfunction and detrimental effects on human health. The nephrotoxicity induced by lead usually occurs in three stages: acute nephropathy; chronic nephropathy; and, finally, renal tubular cell neoplasia or adenocarcinoma [197].

Studies on lead have shown its low mutagenic potential. However, its ability to in-duce oxidative stress and inhibit DNA repair has been reported to enhance the effects of other mutagens, as observed in in vitro studies [198]. Although there is limited evidence linking lead to cancer, several studies have indicated adenocarcinoma, lung, stomach, kidney, and brain cancer as the most probable cancer types associated with lead exposure [197,198,199].

Lead bioaccumulation has also been associated with harmful effects on the immune system, leading to inflammation [24]. It can disrupt immune cells’ production and function, impairing innate and adaptive immune responses. Low levels of lead exposure have been found to stimulate the immune system, while higher levels typically lead to immunosuppression [168]. It has been shown that lead affects the immune system by upregulating the expression of inflammatory mediators and markers, thereby altering immune responses, lymphocyte function, cytokine, and immunoglobulin production [200]. It induces increased production of TNF-α and inflammatory interleukins (IL-1 and IL-6), upregulates expression of COX-1 and COX-2, decreases production of anti-inflammatory IL-10, and enhances thromboxane B2 as well as prostaglandin E2 concentrations in macrophages [24]. In addition, lead affects the IL-2 cytokine, crucial for the growth, proliferation, and differentiation of T lymphocytes, and also adversely affects the IL-4 cytokine, which plays a significant role in the function of B lymphocytes. Since lead can potentially disrupt the balance of the oxidant–antioxidant system and induce oxidative stress, it can trigger inflammatory reactions in multiple organs [201,202].

It has been shown that lead exposure is associated with risk factors for CVD development. A population-based study conducted with 4452 Malmö Diet and Cancer Study (MDCS) participants shows that even low-level lead exposure increases blood pressure and may increase the risk of hypertension [203]. It is supported by the United States National Health and Nutrition Examination Survey (NHANES) study, where a significant association between the blood lead level and hypertension was demonstrated [204]. A newly published cross-sectional study using data from the population-based Swedish CardioPulmonary bioImage Study (SCAPIS) [205], including 5622 middle-aged men and women, found blood lead levels to be associated with an 8% increased risk of carotid artery plaque, as a predictive marker of clinical atherosclerosis. These results are in line with other studies showing an association between body lead levels and different markers of atherosclerosis, like coronary artery calcification [206] and intima–media thickness (IMT) [207]. The systematic review and meta-analysis of Chowdhury et al. [208] explored the link between cardiovascular disease risk and toxic metals. They found that the pooled relative risk (RR) for the lead was 1.43 (95% confidence interval 1.16 to 1.76) for cardiovascular disease, 1.85 (1.27 to 2.69) for coronary heart disease, and 1.63 (1.14 to 2.34) for stroke.

### 4.2. Cadmium

Cadmium is a heavy metal that, when consumed through food and beverages, can exhibit detrimental effects on the nervous system, even at low-dose exposure. The food with the highest cadmium concentration is seafood (0.002–0.644 mg/kg), particularly shellfish, as well as cereals and cereal products, leafy greens, nuts and pulses, starchy roots or potatoes, and meat and meat products. Moreover, cigarettes (1.56 to 1.96 μg of cadmium per cigarette) and alcoholic drinks such as wine (0.10 to 15.38 μg/L), beer (0.80 μg/L), whiskey, gin, and other beverages often contain significant amounts of cadmium due to ethanol contamination during its production [209]. Therefore, strictly controlling this element in food and beverages is highly advisable. The ML values for cadmium span from 0.005 mg/kg in milk protein-based infant foods to 3 mg/kg in supplements. Cadmium concentrations in fruit, vegetables, and fungi are regulated so as to not exceed 0.02–0.5 mg/kg, whereas limits range from 0.05 to 0.15 mg/kg for most meat and fish products. The toxicological guidance value for cadmium presented as PTMI is 25 µg/kg bw [32,33].

The human body has no endogenous clearance mechanism for cadmium, so it accumulates in organisms with a half-life of up to 23.5 years [210]. After absorption by the intestinal epithelium, it enters the systemic circulation, and calcium ATPases and zinc exporters mediate its transport to the nervous system [211]. Numerous in vitro and in vivo studies have confirmed that it enters the neurons and glial cells through voltage-gated Ca^2+^ channels, accumulates, and exhibits a significant impact on the functioning of the peripheral nervous system (PNS) and CNS [212,213]. The vulnerability of the CNS to sustained exposure to low-dose cadmium is particularly concerning, with the developmental brain being especially susceptible to its neurotoxic effects. Initial evidence linking it with neurotoxic effects was documented in the 1980s from children with neurological and learning disabilities who had significantly higher levels of cadmium in their hair in contrast to healthy controls [214]. At the same time, other studies showed that cadmium exposure is associated with an increased risk of peripheral neuropathy, poor performance on visuomotor tasks, and reduced concentration and cognitive function in older adults [215]. In subsequent years, numerous clinical studies and meta-analyses substantiated the association between its levels and neurotoxic effects on the nervous system. These studies have revealed significant correlations between elevated concentrations of cadmium in blood, urine, and cerebrospinal fluid among patients diagnosed with various neurodegenerative diseases, including Alzheimer’s disease, Parkinson’s disease, and amyotrophic lateral sclerosis [212,216,217]. The primary neuronal targets for cadmium-induced toxicity in these neurodegenerative disorders are cerebral cortical neurons [218]. Within these cells, cadmium disrupts cytoarchitecture by affecting the actin and microtubule networks, causing the destruction of microtubules and promoting neurodegeneration and apoptosis [219].

Recent research has indicated that the levels of cadmium that are hazardous to the nervous system in adults are concentrations of >0.8 μg/L in the urine and >0.6 μg/L in the blood [219]. In children, these concentrations are lower (>0.38 μg/L in the blood and >0.1802 μg/L in the urine), and this is explained by the fact that their blood–brain barriers (BBBs) are not yet fully developed and have higher permeability for toxins such as cadmium and other heavy metals. Several proposed molecular mechanisms stand behind the cadmium-induced neurotoxic effect. They involve the induction of oxidative stress, disruption of essential enzyme activity in the nervous system, and alteration of the homeostasis of similar bioelements, such as Ca^2+^, Mg^2+^, and Zn^2+^, that are included in neural transmission in nervous tissue [220]. Rodent experiments have shown that cadmium exposure triggers oxidative stress and targets mitochondria that generate ROS and increase lipid peroxidation in the parietal cortex, striatum, and cerebellum, thus provoking energy deficits and apoptosis [221]. Furthermore, preclinical data have shown that oxidant-induced cascades lead to the destruction of the BBB architecture and its increased permeability in newborn and young rats compared to adults [222]. As a result, this can lead to cellular dysfunction and cerebral edema.

Furthermore, cadmium competes with Ca^2+^ at the voltage-dependent Ca^2+^ channels, disrupting proper neurotransmitter release and altering neurotransmitter signaling [223], thus affecting glutamate, acetylcholine, GABA, and DA neurotransmitter receptor functions in the brain [223]. Animal studies show that it disturbs neurotransmitter homeostasis by reducing excitatory neurotransmitters such as glutamate and aspartate levels and increases the amygdala’s inhibitory neurotransmitters, like glycine and GABA [223]. On the other hand, cadmium can profoundly disrupt glycogen metabolism (extensively reviewed in [215]), alter DNA methylation, and dysregulate gene expression, causing severe DNA damage and genotoxicity [220,224].

Cadmium toxicity has a significant impact on the kidneys, making them one of the most affected organs [225]. Chronic exposure to cadmium, often through contaminated food, can lead to renal dysfunction and damage. Over time, tubular cells accumulate the majority of cadmium absorbed through the gastrointestinal and respiratory systems, and its content in the kidneys is correlated with the quantity assimilated from external sources over an individual’s lifetime [226].

As comprehensively discussed by Hernández-Cruz and coworkers, the key targets for cadmium toxicity are mitochondria. The entry of cadmium into mitochondria results in mitochondria dysfunction, oxidative stress, and apoptosis. Cadmium promotes the generation of ROS by enhancing the activity of nicotinamide adenine dinucleotide phosphate hydrogen (NADPH) oxidase (NOX) and reducing the activity of antioxidant enzymes such as superoxide dismutase (SOD), catalase (CAT), and glutathione peroxidase (GPx). In addition, it increases the production of hydroxyl radicals (OH) by elevating free iron (Fe^2+^) levels. Moreover, cadmium impairs the function of tricarboxylic acid cycle (TCA) enzymes and causes damage to mitochondrial DNA [227]. It has been suggested that the accumulation of cadmium in cytosolic and mitochondrial compartments induces changes in the functioning of kidney proximal tubular epithelial cells. The initial impacts include oxidative stress, cell signaling cascade disruption, and cell adhesion alterations. If the damage surpasses the cellular repair capacity, cell death occurs through apoptosis, necrosis, or a combination of both. As epithelial cells align along the tubular basement membrane via local adhesion molecules to collectively facilitate filtration and reabsorption, increased cell death in this area can result in dissociation among cells and the basement membrane. Consequently, these alterations in epithelial cells may lead to proteinuria, polyuria, and the progressive deterioration of kidney function [228]. Finally, nephrotoxicity caused by prolonged exposure to elevated cadmium levels can progress to CKD, with prevalence rising worldwide [225].

Cadmium exhibits cytotoxic effects and can potentially act as a carcinogen when inhaled, while evidence for its carcinogenic activity through oral ingestion is limited. Some studies have reported that long-term exposure to cadmium through the diet is associated with an increased risk of melanoma and prostate and gastric cancer [229,230,231]. Some previous studies have indicated that airborne cadmium, as a metalloestrogen, can function as an endocrine disruptor, increasing the risk of breast cancer [232]. However, considering dietary exposure, no increase in the risk of breast cancer in women was identified [233]. It was reported that airborne cadmium exposure can lead to increased effective doses. When it enters the lungs, 10% to 50% of the inhaled dose enters the bloodstream [233]. On the contrary, cadmium absorption through the gastrointestinal tract is approximately 6% and can be affected by nutritional factors, such as iron levels [234].

In general, the main cadmium carcinogenic mechanisms involve inflammation, generation of ROS, epigenetic changes, DNA damage, impaired DNA repair, oxidative stress, alterations in gene expression, and abnormal DNA methylation [235]. Impairment of DNA repair mechanisms due to cadmium exposure can lead to the accumulation of damaged DNA, promoting carcinogenesis [168].

As a result of long-term exposure, cadmium accumulates in immune cells and affects the immune system, causing various health issues. It acts as an immunotoxic agent by controlling immune cell activity and apoptosis, changing immune cytokine secretion, triggering ROS production and oxidative stress, modifying the frequency of T lymphocyte subsets, and affecting the production of selective antibodies in immune cells [236]. Cadmium causes inflammation in immune cells by activating several signaling pathways, such as NF-κB and mitogen-activated protein kinase (MAPK) pathways, thus inducing the upregulation of inflammatory mediators and markers [237]. Cadmium exposure affects innate immunity by reducing macrophages’ phagocytic capacity, proliferation, and status transformation. It also decreases the number of natural killer (NK) cells and increases the number of neutrophils, leading to an inflammatory response [236]. Regarding adaptive immunity, cadmium exposure induces apoptosis of T-cells and B-cells. Therefore, it exhibits immunosuppressive effects on innate and adaptive immunity, impairing immune system functions and predisposing individuals to various chronic diseases.

Exposure to cadmium can cause serious cardiovascular problems. The systematic review of Martins et al. [238] examined the correlation between cadmium levels and blood pressure or hypertension. They found a positive association in many studies across various settings, but also noted some studies suggesting an inverse relationship. However, a recent meta-analysis by Aramjoo et al. [239] covering 23 studies confirmed an association between cadmium levels and increased systolic blood pressure and/or diastolic blood pressure and/or hypertension. Further, their findings indicate that the cadmium level in the hair is the optimal biomarker for analyzing the association between cadmium and blood pressure in both genders.

In the cross-sectional study of a large population-based sample from MDCS, the authors [240] observed that blood cadmium concentrations were associated with both the prevalence and size of atherosclerotic plaques in the carotid artery. Comparing quartile 4 with quartile 1 of blood cadmium, and after additional adjustment for all risk factors and predictors of cardiovascular disease, the authors found a 30% higher risk for the prevalence of any plaque. These findings align with the conclusion of Barregard et al. [241], who found cadmium to be associated with coronary artery calcium score performing cross-sectional among SCAPIS participants [242]. Additionally, similar conclusions have been drawn from the meta-analysis of Tinkov et al., who found that high cadmium exposure was associated with atherogenic changes in the lipid profile (total cholesterol, higher LDL, lower HDL) [243]. Eight epidemiological studies dealing with the association of cadmium and cardiovascular disease, summarized in meta-analysis [208], found that relative risks for cadmium were 1.33 (1.09 to 1.64) for CVD, 1.29 (0.98 to 1.71) for CHD, and 1.72 (1.29 to 2.28) for stroke. A prospective cohort study sub-grouped from the Strong Heart Study (SHS), a large population-based cohort study of 13 American Indian communities, was also used to assess the relationship between cadmium exposure and PAD. The results have shown that cadmium is associated with incident PAD, even after adjustment for tobacco consumption, which is of great relevance considering that tobacco is one of the primary contributors to cadmium exposure, demonstrating cadmium’s significance as a CVD risk factor [244]. Additionally, cadmium has been found as a potential quantitative risk factor for the graded development of PAD [245].

### 4.3. Arsenic

Arsenic is a toxic metalloid that exhibits various adverse effects on health, even in small concentrations. Over 200 million people worldwide are chronically exposed to this element, mainly through contaminated groundwater. This applies to low-income developing countries such as Bangladesh, India, Mexico, Thailand, and Latin America [246]. Besides water, arsenic may be present in food; the highest concentrations have been found in fish (16.3–38.1 mg/g), shellfish, rice (0.097–0.25 mg/kg), milk (0.0002–0.05 mg/kg), and cereals (55 µg/kg–158 µg/kg), whereas the lowest were observed in pulses, vegetables, and fruits [247,248]. The levels of arsenic in food mainly depend on the groundwater quality, i.e., its contamination with arsenic when used for crops and plant cultivation. Arsenic testing primarily targets rice-based products, baby foods, fruit juices, and salt. The regulated arsenic content in these products varies from 0.01 to 0.5 mg/kg. BMDL0.5 for arsenic is 3.0 μg/kg bw per day (2.0–7.0 μg/kg bw per day based on the range of estimated total dietary exposure) [32,33].

Upon ingestion, like with other metals, arsenic is adsorbed in the gastrointestinal tract and delivered via blood to the brain. There, it easily crosses the BBB and mainly accumulates in the striatum and hippocampus [249], exhibiting its toxicity and damaging the nervous system. Epidemiological studies indicate that it is a developmental neurotoxicant responsible for intellectual and cognitive impairments in humans, especially among children [250,251]. Namely, Tolins and colleagues reviewed 17 studies. They found that impaired behavior and deficits in cognition and IQ were associated with arsenic exposure, regardless of whether it was acute or chronic [250]. Studies conducted in Bangladesh, India, Mexico, and Taiwan revealed that even low doses of arsenic (<10 g/L) ingested through water during the early prenatal period are associated with low IQ scores and disturbed long-term memory and learning ability [250]. In certain instances, these alterations manifested later in life, during adolescence, and in elderly individuals [250]. However, it is important to highlight that a recent systematic review by Hen and colleagues did not find an association between maternal and prenatal exposure to arsenic and neurodevelopmental impairments in some studies [252]. On the other hand, they showed a clear negative association between arsenic levels and at least one impaired domain of neurodevelopment (executive functioning, general cognition, language, fine motor, or gross motor domains) [252].

Over the years, extensive animal research has proposed several plausible molecular mechanisms for arsenic-induced neurotoxic effects. Primarily, exposure to arsenic causes oxidative stress damage via reduced antioxidant enzymes and increased production of ROS and lipid peroxidation in the brain [253,254,255]. Oxidative stress further leads to structural changes in the brain by changing the essential cellular macromolecules, such as lipids, carbohydrates, and DNA [256]. Additionally, arsenic interacts with SH proteins, disrupts homeostasis of intracellular Ca^2+^ and ATP production, alters membrane potential, triggers neuroinflammation, induces changes in cytoskeletal morphology, and ultimately leads to neuronal apoptosis [253,257]. Altogether, mitochondria, as the primary energy production (ATP) and ROS generation source, may represent the main target of its toxicity [258]. Further on, mitochondrial dysfunction and lack of energy can be associated with the onset of various neurodegenerative disorders, such as Alzheimer’s disease and amyotrophic lateral sclerosis, as neurons and glial cells are highly energy-dependent [259,260]. For example, it has been shown that dimethylarsenic acid (DMA), a metabolite of As in humans, can increase β-amyloid levels and the phosphorylation of Tau protein, an essential feature of Alzheimer’s disease [261,262]. Indeed, a clinical study reported a positive correlation between the levels of increased urinary arsenic excretion and the enhanced risk of progression of Alzheimer’s disease [263]. Moreover, arsenic inhibits the translocation of neurofilaments and reduces its content in peripheral nerves (sciatic nerve), thereby destabilizing the cytoskeletal network, which is the hallmark feature of amyotrophic lateral sclerosis [264,265]. In addition to neurons, arsenic increases proinflammatory cytokine levels in astrocytes, a type of glial cell, potentially leading to elevated levels of amyloid precursor protein (APP) and subsequent α-synuclein aggregation, known as a molecular signature of Parkinson’s disease [246,266].

Aside from changes in oxidative metabolism, rodent studies indicate that low and moderate arsenic exposure can significantly damage different neurotransmitter systems. It has been shown to interfere with glutamatergic, GABA serotonergic, cholinergic, and dopaminergic systems, resulting in altered behavior and impaired synaptic transmission [16,253]. Arsenic predominantly affects glutamate signaling, specifically inducing intricate changes in NMDAR subunit expression within the hippocampus in animal models [16]. These effects involve either upregulation or downregulation of subunit expression, contingent upon the timing of arsenic exposure during neurodevelopment [16]. Electrophysiological studies in arsenic-exposed rodent models have shown alterations in long-term potentiation (LTP) and synaptic plasticity [246]. Disruption of these processes was followed by cognitive deficits, which manifested in impaired learning and memory [267,268]. Aside from glutamatergic receptors, arsenic can affect the development of the cholinergic and dopaminergic systems, particularly through down-regulation of the α7 nicotinic receptors and DA-D2 receptors in rats’ brains [269].

Studies have shown that arsenic exposure increases the risk of CKD, decreases the estimated GFR, and can cause proteinuria and kidney cancer in some cases [270]. Animal studies and in vitro investigations have demonstrated the nephrotoxic effects of arsenic, including vacuolation of tubular cells, interstitial nephritis, and glomerular enlargement. In rats, exposure to arsenic has been linked to increased production of ROS and nitrogen species, which leads to oxidative stress and damage to cellular structures and functions [271]. In a recent study, Chen and coworkers discovered that arsenic exposure led to changes in kidney proteins, particularly in the mitochondrial membrane, oxidative phosphorylation, and the electron transport chain [272]. These protein alterations cause mitochondrial dysfunction, oxidative stress, apoptosis, and autophagy, all linked to kidney injury. Moreover, arsenic exposure increased the expression of bicarbonate transporters in proximal tubules, possibly in response to arsenic-induced uric acid imbalance [272].

Arsenic is classified as a Group 1 human carcinogen, and its carcinogenic effects are believed to involve DNA damage, interference with DNA repair mechanisms, and the promotion of tumor formation [272]. Chronic exposure to arsenic, often through contaminated water and food, is associated with an increased risk of various cancers, including skin, lung, bladder, and liver cancers [273]. Oxidative stress, chromosomal abnormalities, disrupted DNA synthesis and repair, alteration of cellular signaling via changes in activation, expression, and DNA binding activity of transcription factors, abnormal gene expression, and alteration in the expression of growth factors are potential mechanisms in arsenic-induced cancer development [273]. Several studies have reported that populations exposed to arsenic may develop liver lesions, including hepatocellular carcinoma and angiosarcoma [168,274].

An increasing body of evidence suggests that arsenic can harm the immune system, though its exact targets for immune dysfunction are poorly understood. Inorganic arsenic compounds like arsenite and arsenate are highly toxic to macrophages and lymphocytes. In contrast, arsenic trioxide exhibits important anti-tumor properties, particularly against promyelocytic leukemia, lung cancer, and colon cancer [275]. Arsenic affects various aspects of the immune system, including T-cell activation, cytokine expression (e.g., interleukins, interferon-gamma, TNF-α), granulocyte–macrophage colony-stimulating factor, and contact hypersensitivity responses [276]. Chronic exposure to arsenic, especially in early life, can impair both humoral and cellular immune responses, increasing the risk of infections and inflammatory diseases [277]. Arsenic also induces the overexpression of keratinocyte-derived growth factors, likely contributing to skin hyperkeratoses and cancer [278]. Chronic arsenic exposure disrupts gene expression and regulation, leading to immunotoxicity [277].

The association between arsenic exposure and cardiovascular diseases has been extensively studied, with results showing a significant link between arsenic exposure and an increased risk of cardiovascular conditions such as hypertension, atherosclerosis, and heart disease. A systematic review and dose–response meta-analysis by Zhao et al. [279], which included 23 cross-sectional studies, found a pooled odds ratio of 1.14 (95% CI: 1.06, 1.23) for arsenic exposure and prevalence of hypertension, which is in line with the previous results of Abhyankar et al. [280] and has been additionally confirmed by the Strong Heart Family Study (SHFS) study of Kaufman et al. [281]. Data from the sub-grouped SHS showed urine arsenic to be positively associated with carotid IMT and increased plaque score. A cross-sectional study from Bangladesh adults consistently found a positive association between past urinary arsenic levels and carotid IMT [282]. A recent cross-sectional study [283] conducted in 1570 hypertensive adults from the NHANES found significant associations between urine arsenic concentrations and predicted 10-year atherosclerotic cardiovascular disease risk in men. Further, the meta-analysis, which included 12 primary studies, found a link between cardiovascular disease risk and arsenic levels [208]. Comparing the top versus bottom thirds of baseline levels, the authors found that the pooled relative risk for arsenic was 1.30 (1.04 to 1.63) for CVD, 1.23 (1.04 to 1.45) for CHD, and 1.15 (0.92 to 1.43) for stroke. Additionally, the data from SHS have also been used to evaluate whether arsenic exposure is linked to PAD. 

### 4.4. Nickel

Nickel is present in food primarily due to leaching from kitchen utensils and the natural accumulation of nickel in food plants from the soil [284]. While the exact contribution from kitchen utensils is unclear, it is speculated that this could add as much as 1 mg of nickel per day to the diet [285]. Certain foods, notably chocolate, are known for containing high levels of nickel (159.75 μg per 100 g) [286]. Also, cocoa tops (107.96 μg per 100 g) [287], soybeans (347.34 μg per 100 g) [288], and oatmeal (120 μg per 100 g) [289] have been shown to have high concentrations of nickel.

Nickel is present in various oxidation states, with the divalent form being the most stable in food and drinking water. The bioavailability of ingested nickel in humans depends on factors such as the solubility of the nickel compound, the dosing vehicle, and whether the individual is fasting [290]. Absorption is low when nickel is consumed with food or under non-fasting conditions (0.7–2.5%). In contrast, higher absorption rates (25–27%) are reported when ingested in drinking water or under fasting conditions [290]. However, the number of participants in relevant human studies has been limited, and there is significant variability among individuals [290]. Epidemiological studies examining the relationship between nickel exposure and kidney function are scarce and yield conflicting results. Some studies have provided evidence for the role of nickel in renal impairment. A recent study by Nan and coworkers showed that nickel exposure is associated with decreased kidney function [291]. Similarly, a significant correlation between blood nickel levels and decreased estimated glomerular filtration rate (eGFR) was also reported [292].

Oxidative stress is a mechanism of nickel toxicity whereby the formation of nickel–mercaptan complexes can disrupt the balance of glutathione reductase and the mitochondrial antioxidant defense system [293]. Nickel may cause DNA damage, inhibit repair mechanisms, and block methylation [294]. Through repeated or chronic exposure, nickel can induce damage to renal tubular epithelial cells, leading to kidney disease and raising the risk of end-stage renal disease [295]. Nickel-exposed renal tissue in mice shows tubular injury with inflammation and focal damage primarily at the corticomedullary junction [296]. However, despite these findings, there is limited understanding of the long-term effects of nickel on human kidneys and overall health [294].

Nickel compounds exhibit low activity in bacterial mutagenicity tests and only weak mutagenicity in cultured mammalian cells [290]. However, at high, cytotoxic levels, nickel compounds can induce oxidative DNA damage, DNA single-strand breaks, DNA–protein cross-links, sister chromatid exchanges, chromosomal aberrations, and micronuclei in mammalian cells, likely through both aneugenic and clastogenic mechanisms [297,298]. Dysregulation of signaling pathways and changes in chromatin structure may also contribute to nickel genotoxicity. Animal studies have shown no tumors following oral administration of soluble nickel compounds [299]. Also, there are no available data linking cancer in humans to oral nickel exposure. Nickel is classified as a human carcinogen through inhalation exposure, but no evidence links human cancer to dietary nickel exposure [290].

It has been shown that nickel affects the immune system, acting as a sensitizer that can lead to hypersensitivity reactions upon exposure. While oral exposure to nickel is not known to cause sensitization, it can trigger eczematous flare-ups in individuals already sensitized to nickel and suffering from systemic contact dermatitis (SCD) [299]. However, there are uncertainties regarding adverse reactions after nickel ingestion, as studies have involved a limited number of participants with varying degrees of sensitivity, and the outcomes have been reported in different ways, making comparisons challenging [290]. The ability of nickel to bind to proteins induces specific immune responses that lead to allergic reactions, which can manifest in the skin or elsewhere in the body [300]. Nickel also has non-specific immune activity, inducing inflammatory reactions through Toll-like receptors (TLRs) and NF-κB signaling pathways, possibly contributing to adverse reactions [301]. While skin exposure is the most common route for nickel reactions, oral exposure can induce similar effects, particularly in sensitized individuals with SCD [290]. Nickel may also disrupt the immune system by causing apoptosis of monocytes, as demonstrated in vitro, potentially affecting host resistance [302].

### 4.5. Mercury

Aquatic microorganisms absorb methylate mercury, creating methylmercury (MeHg), which enters the food chain and reaches the human body through fish consumption [303]. Despite cooking fish, its mercury content remains unaffected [304]. Different types of fish contain varying levels of mercury, which is important to consider for overall health. High-mercury fish, which have levels exceeding 0.3 mg per 100 g, include shark (0.0979 mg per 100 g), swordfish (0.0971 mg per 100 g), king mackerel (0.0730 mg per 100 g), and tilefish from the Gulf of Mexico (0.1450 mg per 100 g). Moderate-mercury fish, with levels ranging from 0.1 to 0.3 mg per 100 g, include various types of tuna, such as albacore and yellowfin, which range from 0.0118 to 0.0391 mg per 100 g, and orange roughy at 0.0571 mg per 100 g. Low-mercury fish, with levels less than 0.1 mg per 100 g, include salmon (typically less than 0.0022 mg per 100 g), shrimp (less than 0.0009 mg per 100 g), catfish (less than 0.0025 mg per 100 g), and pollock (less than 0.0031 mg per 100 g) [305].

Mercury thresholds are established for specific food categories such as salt, supplements, fish, and fishery products. Salt and supplements must not surpass 0.1 mg/kg of mercury, while the ML for fish and fishery products varies by species, ranging from 0.3 to 1 mg/kg. The toxicological guidance value for inorganic mercury presented as PTWI is 4 μg/kg bw [32,33].

Mercury lacks any physiological role in humans, so there are no effective mechanisms to excrete it from the organism. Its accumulation in the body can lead to kidney disease, characterized by proteinuria and toxic encephalopathy. Patients with mercury-related kidney damage often exhibit symptoms such as edema, changes in urine volume, proteinuria, and nephrotic syndrome, along with other common signs of mercury poisoning like excitability, gingivitis, and tremor [306].

The pathogenesis of mercury-induced kidney disease is not fully understood, but immune mechanisms are thought to play a crucial role in glomerular diseases [306]. Mercury combines with proteins, forming haptens, which produce antigen–antibody complexes that can penetrate the glomerular membrane, causing glomerular lesions [307]. Additionally, mercury ions are highly toxic to renal tubules, causing vacuolation, degeneration, and necrosis of tubular epithelial cells, resulting in kidney disease. Moreover, mercury binds to sulfhydryl groups in the body, reducing levels of sulfhydryl-containing enzymes and membrane proteins’ activity and altering the cell membrane structure and function [308]. This process leads to increased ROS levels outside the cell and decreased activity of free radical scavenging systems, causing oxidative stress damage [308].

Although the history of MeHg’s toxic effects on CNS function has long been recognized, two incidents of mass food poisoning during the last century significantly drew public attention. Namely, in Japan and Iraq, there was mass poisoning of people who ingested food and wheat that had previously been contaminated with high amounts of MeHg from industrial waste and fungicide [309,310]. These incidents resulted in multiple neurological impairments with a range of severe symptoms, including ataxia, speech impairment, sensory disturbances, and death. Infants born to affected mothers exhibited various neurodevelopmental and cognitive deficits. Prenatal exposure to MeHg is of particular concern due to its ability to easily cross the placental barrier and BBB and accumulate mainly in the cerebral cortex and cerebellum without efficient clearance compared to adults [311]. Many studies have confirmed that prenatal MeHg exposure is tightly associated with neurodevelopmental deficits in children, including reduced IQ, impaired language skills, and defects in coordination, attention, memory, language, and motor function [312,313,314]. Particularly, the study on children from the Faroe Islands found that prenatal mercury exposure induces defects in attention, memory, language, and motor function [315]. Also, maternal exposure to MeHg during these critical developmental stages is connected with other brain disorders such as autism, ADHD, and mental retardation [316]. Besides developmental diseases, some evidence highlights the link between chronic exposure to MeHg and the early onset of neurodegenerative diseases, such as Parkinson’s and multiple sclerosis [311]. However, it must be noted that in 2022, a systematic review was published that analyzed 32 prospective studies and found that exposure to dietary mercury during pregnancy is unlikely to be a risk factor for low developmental progress during early childhood [317].

At the molecular level, the primary mechanism underlying mercury effects is cytotoxicity, attributed to excessive glutamate and oxidative stress, leading to excessive ROS generation and lipid peroxidation [311,314]. Studies have also indicated that mercury binds with high affinity to SH groups of tubulin, a protein crucial for neurodevelopment, leading to cyto-architecture alterations and neuronal apoptosis [308]. Also, it has been shown that MeHg disrupts neuronal development by interfering with cell migration, dendritic arborization, synaptogenesis, and neuroinflammation in experimental animals [311,318]. Apart from neurons, research shows that mercury-induced damage to cytoskeleton proteins is not restricted to neurons, but also affects other brain cells, such as astrocytes [319]. MeHg also interferes with the intracellular signaling of multiple receptors (e.g., NMDA, GABA, DA, and acetylcholine (ACh) receptors) and promotes the blockade of Ca^2+^ channels in neurons [16]. For example, in vivo studies in rats have shown that exposure to MeHg over-activates the NMDA receptors in cortical neurons in the developing and adult brain, inducing neurotoxicity that causes severe DNA damage [320,321].

Mercury negatively affects the immune system by promoting autoantibody production, inhibiting T lymphocyte function, and inducing autoimmune diseases [322]. As has been comprehensively discussed by Pollard and coworkers, several studies comprising fish consumers have indicated significant associations between mercury exposure levels and increased expression of cytokines, such as IL-6, IFN-γ, IL-4, and IL-17. However, some studies have not shown consistent associations with cytokine levels [307]. Mercury-induced autoimmunity is considered to begin with an inflammatory reaction at the exposure site, which leads to the activation and clonal expansion of T-cells, followed by an increase in lymph node size and the formation of new germinal centers. This process triggers the production of IgG antibodies, specifically IgG ANAs, which form immune complexes in the glomeruli and blood vessels [24]. This inflammation is believed to be caused by tissue damage, which prompts the release of damage-associated molecular patterns that activate innate immune sensors, such as nucleic acid-sensing TLRs, leading to the production of inflammatory cytokines and the induction of chronic inflammation [24]. Chronic tissue injury and inflammation caused by mercury accumulation can lead to secondary lymphoid organ enlargement and the creation of ectopic lymphoid structures [323]. These early inflammatory events, including cathepsin B activation and the release of proinflammatory cytokines, are linked to subsequent autoimmune responses [24]. The observed findings support the fundamental role of mercury exposure in the induction of inflammation and autoimmunity.

Exposure to MeHg may contribute to the development of risk factors associated with cardiovascular disease. Epidemiological studies evaluating the IMT of the carotid artery have found that mercury levels are associated with accelerated progression of carotid atherosclerosis [324,325]. Diverse and conflicting findings regarding the association between mercury exposure and blood pressure were synthesized in the systematic review and meta-analysis of Hu et al. [326]. Their analysis revealed either no significant or a weak association between mercury exposure and blood pressure in cases of low to moderate mercury exposure levels, while also indicating a positive relationship among populations exposed to higher levels of mercury. Additionally, systematic review and meta-analysis by Hu et al. observed the association between mercury exposure and CVDs and all-cause mortality using data from 14 studies including more than 34,000 participants. Their analysis revealed that mercury exposure was linked to an increased risk of nonfatal ischemic heart disease (IHD) (relative risk (RR): 1.21 (0.98, 1.50)), cardiovascular disease mortality (RR: 1.68 (1.15, 2.45)), and mortality from other heart diseases (RR: 1.50 (1.07, 2.11)), as well as all-cause mortality (RR: 1.21 (0.90, 1.62)). The most sensitive cardiovascular endpoint observed was MI or IHD. Additionally, the researchers identified nonlinear dose–response relationships between MeHg exposure and CVD outcomes. Surprisingly, this meta-analysis found no association between mercury exposure and stroke despite the considerable overlap in risk factors for stroke and IHD. The authors suggested that the absence of this association might be attributed to insufficient statistical power due to the limited number of studies reporting stroke as an outcome and the use of different biomarkers to assess mercury exposure.

## 5. Conclusions

Maintaining a balanced dietary intake of essential metals through food is crucial for supporting various body functions and preventing deficiencies that can lead to health issues. When consumed in adequate amounts, essential metals ensure that the body operates efficiently and remains healthy. However, both deficiency and excess intake can lead to serious health problems. On the other hand, food can also be a source of toxic metals which do not play a beneficial role in biological functions and pose significant health risks due to their toxicity. Even low levels of toxic metals in food can cause severe damage to human health, highlighting the importance of controlling exposure through dietary sources. Therefore, minimizing exposure to toxic metals through food is vital, as it ensures that food safety regulations and environmental protections are rigorously applied and monitored. Ensuring the safety of our food supply requires continuous research into contamination pathways and their health impacts. Advanced monitoring technologies and stricter regulatory frameworks are necessary to address the evolving challenges posed by metal contaminants in food. Looking forward, developing innovative solutions to prevent contamination and ensure our food supply’s safety and nutritional quality is essential. Balancing the benefits and risks associated with metal content in food will help to protect public health and ensure a safer, healthier future.

## Figures and Tables

**Table 1:** Types of culinary salts and their sodium content.

Type of Culinary Salt	Sodium Content (mg per Teaspoon)
Iodized salt	2360
Kosher salt	1240
Low-sodium salt	1770
Pink Himalayan salt	1680
Sea salt	2000

**Table 2:** Types of milk and their calcium content.

Type of Milk	Calcium Content (mg per 200 mL)
Semi-skimmed cow milk	240
Skimmed cow milk	244
Whole cow milk	236
Sheep milk	380
Coconut milk	54
Oat milk	16
Almond milk	90

## Data Availability

No new data were created or analyzed in this study. Data sharing is not applicable to this article.
